# Different responses of weather factors on hand, foot and mouth disease in three different climate areas of Gansu, China

**DOI:** 10.1186/s12879-017-2860-4

**Published:** 2018-01-08

**Authors:** Faxiang Gou, Xinfeng Liu, Jian He, Dongpeng Liu, Yao Cheng, Haixia Liu, Xiaoting Yang, Kongfu Wei, Yunhe Zheng, Xiaojuan Jiang, Lei Meng, Wenbiao Hu

**Affiliations:** 1 Institute for Communicable Disease Control and Prevention, Gansu Center for Diseases Prevention and Control, Lanzhou, Gansu China; 2 School of Public Health and Social Work, Institute of Health and Biomedical Innovation, Queensland University of Technology, Brisbane, Queensland Australia

**Keywords:** Hand, foot and mouth disease, Climate change, Generalized linear model, Classification and regression tree

## Abstract

**Background:**

To determine the linear and non-linear interacting relationships between weather factors and hand, foot and mouth disease (HFMD) in children in Gansu, China, and gain further traction as an early warning signal based on weather variability for HFMD transmission.

**Method:**

Weekly HFMD cases aged less than 15 and meteorological information from 2010 to 2014 in Jiuquan, Lanzhou and Tianshu, Gansu, China were collected. Generalized linear regression models (GLM) with Poisson link and classification and regression trees (CART) were employed to determine the combined and interactive relationship of weather factors and HFMD in both linear and non-linear ways.

**Results:**

GLM suggested an increase in weekly HFMD of 5.9% [95% confidence interval (CI): 5.4%, 6.5%] in Tianshui, 2.8% [2.5%, 3.1%] in Lanzhou and 1.8% [1.4%, 2.2%] in Jiuquan in association with a 1 °C increase in average temperature, respectively. And 1% increase of relative humidity could increase weekly HFMD of 2.47% [2.23%, 2.71%] in Lanzhou and 1.11% [0.72%, 1.51%] in Tianshui. CART revealed that average temperature and relative humidity were the first two important determinants, and their threshold values for average temperature deceased from 20 °C of Jiuquan to 16 °C in Tianshui; and for relative humidity, threshold values increased from 38% of Jiuquan to 65% of Tianshui.

**Conclusion:**

Average temperature was the primary weather factor in three areas, more sensitive in southeast Tianshui, compared with northwest Jiuquan; Relative humidity’s effect on HFMD showed a non-linear interacting relationship with average temperature.

## Background

Hand, foot and mouth disease (HFMD), a common viral disease that usually affects children, caused by a group of viruses including enterovirus A71 (EV-A71) and coxsackievirus A16 (CV-A16). Most HFMD cases are mild self-limiting, characterized by fever, and flat spots or bumps on the hands, feet and mouth; severe cases could develop into cardiopulmonary failure and death [1].

HFMD outbreaks have been frequently reported in Asia-Pacific in the past two decades, posing great threats to the public health, including Taiwan (1998), Singapore (2000), South Korea (2000), Vietnam (2005), Hong Kong (2010), Cambodia (2012) and China (2007, 2008) [2–9]. After two severe outbreaks in Linyi City in 2007 and Fuyang City in 2008, HFMD has been classified as a Class III notifiable communicable disease by the Chinese government [8, 9]. But vaccines and antiviral drugs against EV-A71 or CV-A16 are still on the way to limit the spread of HFMD [10, 11].

Many studies have proved that meteorological factors contribute to the epidemic of HFMD, including temperature, relative humidity, rainfall, wind speed and sunshine [12–15]. Meteorological effects on the HFMD differ from place to place. For example, a positive effect of temperature at lag of 5 days, a negative effect of relative humidity at lag of 1 day and a positive effect of relative humidity at lag of 5–7 days were observed on adolescent HFMD in an eastern city of China [12]. However, another study in a southern city of China has shown that temperature was negatively correlated with HFMD following a lag of 1–3 days, turned to be positive at lag of 5–9 days; a positive effect was also observed at lag of 3–10 days [13]. In another study in East Asia reported that the average temperature was not significantly associated with HFMD [15]. Moreover, few studies have fully explored the interacting effects of weather factors on HFMD in different climate regions remain to be evaluated. Thus, in this study, three areas located with different climate features were chosen to determine the linear and non-linear interacting relationship between weather factors and HFMD in children in three different climate areas in Gansu, China, and gain further traction as an early warning signal based on weather variability for HFMD transmission.

## Methods

### Data sources

**Fig. 1:**
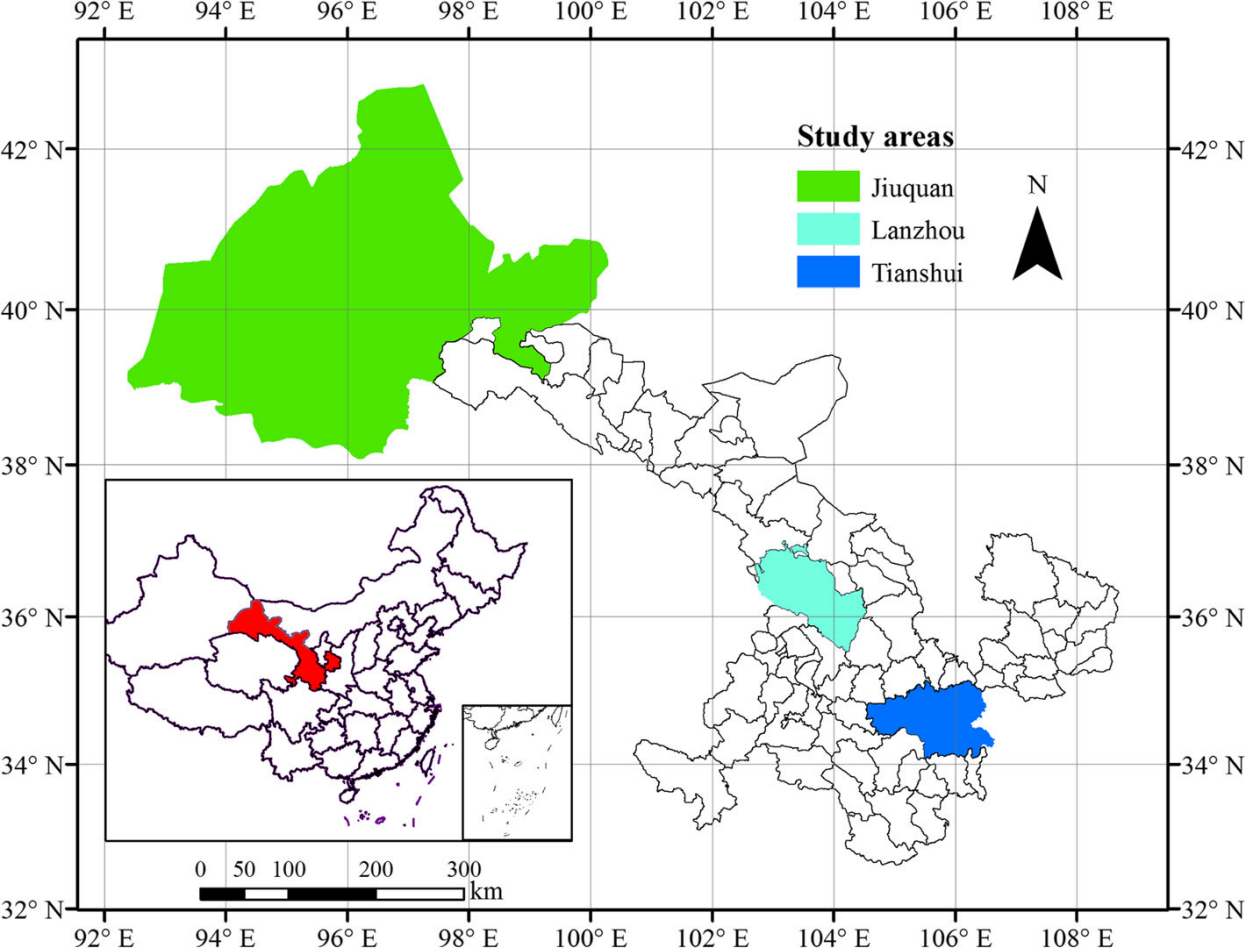
The study areas and their location in Gansu province and China. This figure was generated using ArcGIS software version 10.0 (ESRI, USA)

Gansu is a province in northwestern China, constituting 14 cities. From southeast to northwest, its climate becomes much drier and colder across the province. Three areas, including Tianshui, Lanzhou and Jiuquan, were chosen as the different climate zone (Fig. 1). Tianshui, with a population of 3.7 million, located in the southeast of Gansu, has a climate of continental monsoon influenced, semi-humid and semi-arid climate. In the central Gansu and as the capital city of Gansu, Lanzhou has 3.6 million populations, has a temperate and monsoons climate. Be different from those two areas, Jiuquan in the northwest of Gansu has a cold desert climate, with a population of 1.1 million.

Weekly HFMD cases between 2010 and 2014 were obtained from the national infectious disease surveillance system. Since 2008, HFMD has been included into the surveillance of China Information System for Disease Control and Prevention (CISDCP). All suspected or confirmed HFMD cases should report with the system within 24 h by medical institutions mainly by hospitals. The diagnostic of HFMD was based on the Clinic Guidance of HFMD (http://www.nhfpc.gov.cn/mohyzs/s3586/201004/46884.shtml, in Chinese). Patients diagnosed as HFMD usually by clinical signs and symptoms in epidemic seasons, or atypical cases would test by a throat swab or stool specimen for laboratory diagnosis by culture or polymerases chain reaction.

Weekly weather information, including average temperature (AT), maximum temperature (MaxT) and minimum temperature (MinT), temperature difference (TD, calculated by the difference of MaxT and MinT), relative humidity (RH) and rainfall (RF), which were obtained from the Gansu Meteorological Bureau. Also, population aged less than 15 of each area was obtained from the yearbook of Gansu (2011) (http://data.cnki.net/area/Yearbook/Single/N2012120605?z=D28).

### Statistical methods

#### Seasonal decomposition

Seasonal decomposition was firstly performed to explore the underlying pattern in the HFMD infection, including seasonal, trend-cycle and “error” component using multiplicative model, implemented by ratios-to-Moving Averages method, also known as Census Method I [16].

#### Cross-correlation analysis

Cross correlation analysis was applied to search the most appropriate moving average (MA) of weather factors in each study areas. Cross-correlation analysis is a measure of correlation between two univariate series [17]; the strength of the correlation can be measured by the absolute value of cross correlation function (CCF).

#### Regression models

Generalized linear regression model (GLM) was used to measure the linear relationship of weather factors and HFMD. To further explore the interactive nonlinear effect, Classification and regression tree model (CART) was used to detect the threshold value that had the best ability to split node in the optimal trees. Description about the two algorithms have been accomplished elsewhere [18, 19]. In this study, GLM was fitted with the logarithm as the link function, with the Poisson as the response distribution, adjusted by seasonal components. And when modeling for CART, weather factors including the original and lagged moving average weather factors were involved in, and complexity parameter was used for pruning tree.

Descriptive statistics, chi’s square test, seasonal decomposition, GLM and CART analysis were applied by R 3.1.2 (http://www.r-project.org/) [20].

## Results

### Descriptive statistics

Descriptive statistics of weekly HFMD cases and weather factors among three study areas in 2010–2014 were presented in Table 1. Mean weakly HFMD incidence of Lanzhou was 12.31/100000, which was greater than other two areas. The mean of weekly AT, MaxT, MinT, RH and RF increased, but TD decreased from northwest Jiuquan to southeast Tianshui in 2010–2014 (Table 1). No death HFMD cases in three study areas was reported during the study period.

**Table 1:** Descriptive statistics of weekly HFMD and weather factors among three study areas in 2010–2014

	Variables	Minimum	1st Quartile	Median	3rd Quartile	Maximum	Mean	Std. deviation
Jiuquan	HFMD	0.00	1.00	7.00	21.50	141	16.55	24.27
	Incidence (/100,000)	0.00	0.54	3.80	11.66	76.45	8.97	13.16
	AT (°C)	−15.81	−2.08	10.54	19.31	29.93	8.49	11.47
	MaxT (°C)	−11.61	5.28	17.96	26.28	35.31	15.59	11.62
	MinT (°C)	−20.07	−8.25	2.93	12.12	20.40	1.84	10.96
	TD (°C)	6.90	12.44	13.87	15.20	17.71	13.75	2.04
	RH (%)	15.71	32.57	43.36	52.18	81.14	42.97	13.74
	RF (mm)	2.00	4.00	4.00	6.00	44.14	7.56	9.41
Lanzhou	HFMD	0.00	9.00	31.50	77.25	429	58.48	72.10
	Incidence (/100,000)	0.00	1.89	6.63	16.26	90.28	12.31	15.17
	AT (°C)	−8.87	1.83	12.87	19.97	29.76	11.08	9.98
	MaxT (°C)	−3.66	8.46	19.55	26.32	37.56	17.74	10.29
	MinT (°C)	−13.40	−2.78	7.11	14.57	23.26	5.94	9.52
	TD (°C)	6.30	10.05	11.90	13.48	18.06	11.80	2.40
	RH (%)	19.14	43.36	52.29	60.00	78.43	50.95	12.47
	RF (mm)	1.57	4.00	4.86	24.57	82.57	13.91	16.65
Tianshui	HFMD	0.00	2.00	10.00	28.25	222	24.55	36.80
	Incidence (/100,000)	0.00	0.30	1.51	4.27	32.64	3.61	5.41
	AT (°C)	−5.59	3.63	13.39	20.11	26.61	12.09	8.84
	MaxT (°C)	−0.61	9.68	19.40	25.69	33.67	18.01	9.15
	MinT (°C)	−10.11	−0.56	8.54	15.59	21.61	7.54	8.71
	TD (°C)	2.86	8.29	10.51	12.49	16.73	10.47	2.75
	RH (%)	31.00	56.54	65.00	72.14	88.57	63.55	11.49
	RF (mm)	2.14	4.14	10.36	29.07	99.14	18.01	20.07

*AT* average temperature, *MaxT* maximum temperature, *MinT* minimum temperature, *TD* temperature difference, *RH* relative humidity, *RF* rainfall

### Sequence charts comparison of weekly HFMD and weather factors among study areas

**Fig. 2:**
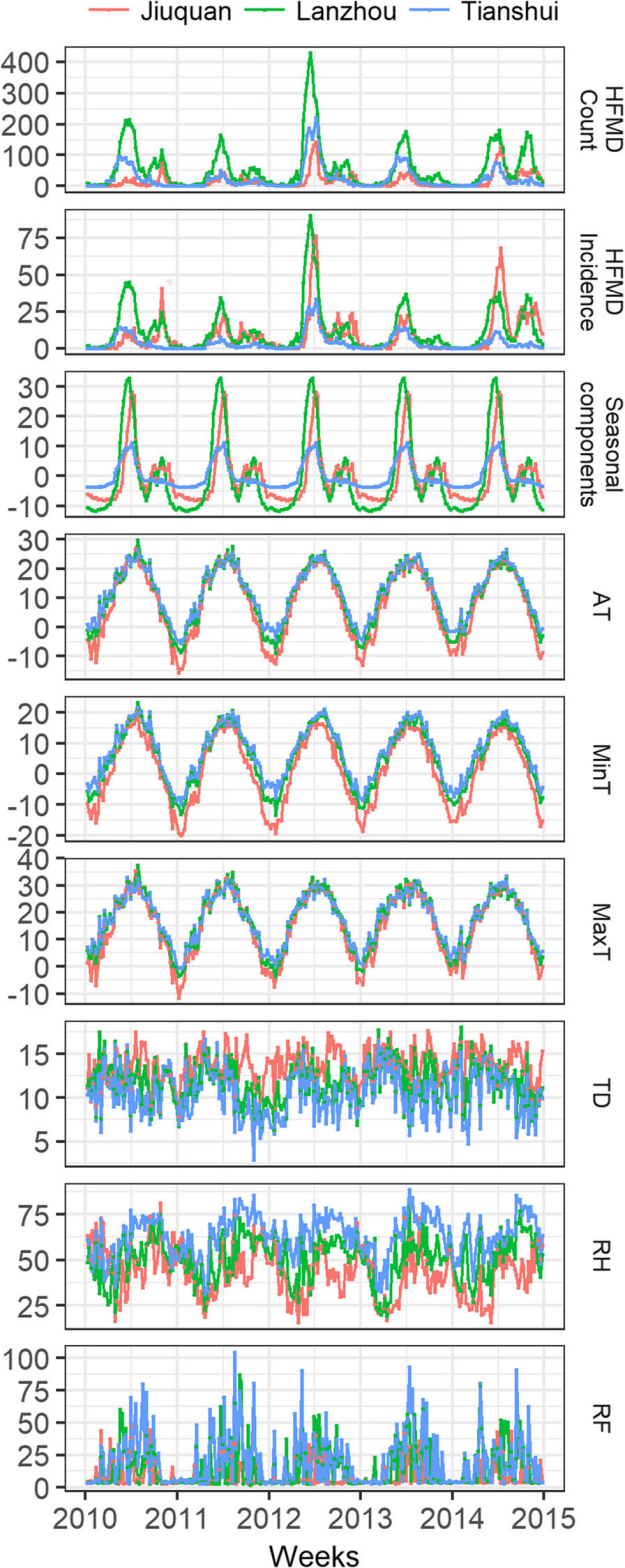
Sequence chart of weekly HFMD and weather factors among three study areas in 2010–2014. AT: temperature; MaxT: maximum temperature; MinT: minimum temperature; TD: temperature difference; RH: relative humidity; RF: rainfall

As shown in Fig. 2, weekly variations of HFMD and weather factors in three study areas have similar but different seasonality. Specially, peaks of weekly HFMD counts and incidence did not occur at the same week each year as weather factors did; AT, MaxT and MinT in Lanzhou and Tianshui got their peaks a little later than weekly HFMD count and incidence. Seasonal components in three areas were extracted from weekly HFMD incidence time series using seasonal decomposition for seasonality analysis purpose. And the result showed that main peaks of seasonal components in Lanzhou (25th week yearly) was earlier than which in Jiuquan (27th week yearly), and Tianshui (27th week yearly), and the secondary peaks in Jiuquan (40th week yearly) and Lanzhou (44th week yearly) were much more obvious than which in Tianshui.

### Bivariate correlation between weekly HFMD and weather factors among study areas

**Fig. 3:**
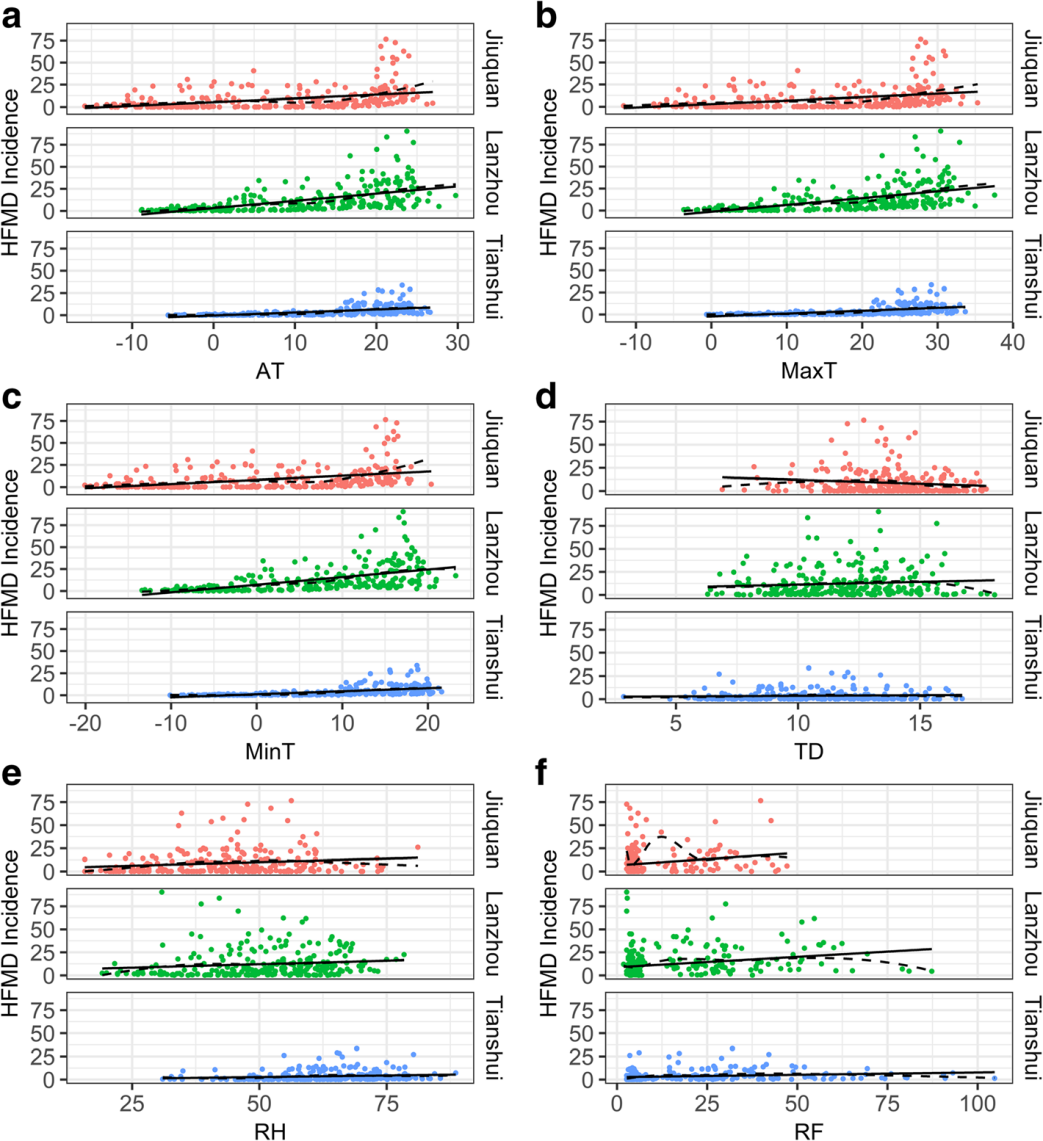
Scatter plot of weekly HFMD incidence and weather factors among three study areas in 2010–2014. A = AT: temperature; B = MaxT: maximum temperature; C = MinT: minimum temperature; D = TD: temperature difference; E = RH: relative humidity; F = RF: rainfall

Figure 3 showed that AT, MaxT, MinT, RH and RF were positively associated with weekly HFMD incidence, while TD showed inconsistent association with HFMD incidence among study areas. HFMD incidence increased with TD in Lanzhou and Tianshui, but decreased in Jiuquan.

**Fig. 4:**
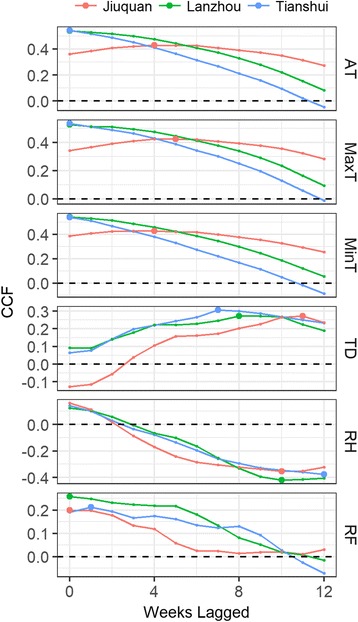
Cross-correlation analysis between weekly HFMD and weather factors among three study areas in 2010–2014. AT: temperature; MaxT: maximum temperature; MinT: minimum temperature; TD: temperature difference; RH: relative humidity; RF: rainfall. CCF: cross correlation function

Cross-correlation analysis also revealed that weekly HFMD infection had highest CCF with weather factors that lagged 0 week, except for TD and RH, which got their highest CCF with weekly HFMD when factors lagged about 7–12 weeks, respectively (Fig. 4). And it should also be noticed that the numbers of lagged week of weather factors had highest CCF with weekly HFMD appeared to slightly different in three study areas (showed as the biggest point in each line). Taking AT as an example, AT in Jiuquan got its highest CCF with weekly HFMD when AT lagged 4 weeks, while in Lanzhou and Tianshui AT got their highest CCF with weekly HFMD with the original series (lagged 0 week).

### Generalized linear model

**Fig. 5:**
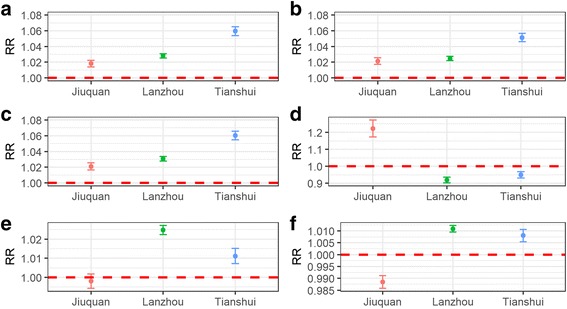
Relative risks of moving average of weather factors on HFMD among three study areas in 2010–2014. A = AT: temperature; B = MaxT: maximum temperature; C = MinT: minimum temperature; D = TD: temperature difference; E = RH: relative humidity; F = RF: rainfall. RR: relative risk

As suggested by GLM, moving average of AT, MaxT and MinT showed similar association with weekly HFMD in three study areas, which was different from TD, RH and RF (Fig. 5). With 1 °C increase of AT, an increase in weekly HFMD of 5.95% [95% Confidence Interval CI): 5.38%, 6.53%] could be observed in Tianshui, compared with 2.80% [2.52%, 3.09%] in Lanzhou and 1.82% [1.39%, 2.24%] in Jiuquan. MaxT and MinT also revealed similar associations with weekly HFMD in three study sites. With 1 °C increase of TD, our results from GLM suggested an increase in weekly HFMD of 22.21% [7.36%, 27.26%] in Jiuquan, but decrease by 8.16% [6.50%, 9.80%], 5.12% [3.24%, 6.95%] in Lanzhou and Tianshui, respectively. The GLM also demonstrated that with 1% increase of RH could increase weekly HFMD by 2.47% [2.23%, 2.71%] and 1.11% [0.72%, 1.51%] in Lanzhou and Tianshui, respectively. No association between RH and HFMD was observed in Jiuquan. RF’s effect on HFMD in three study sites was similar with that of RH. With 1 mm increase of RF, a decrease in weekly HFMD of 1.15% [0.89%, 1.42%] in Jiuquan, but increase in weekly HFMD of 1.09% [0.95%, 1.23%] and 0.80% [0.55%, 1.06%] in Lanzhou and Tianshui respectively.

### Classification and regression tree model

**Fig. 6:**
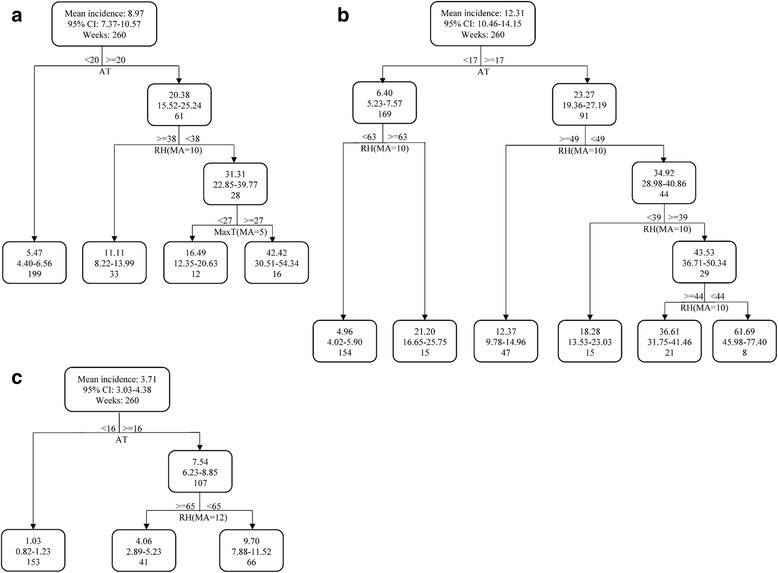
CART modeling the relationship of weekly HFMD incidence and weather factors in 2010–2014. AT: temperature; MaxT: maximum temperature; MinT: minimum temperature; TD: temperature difference; RH: relative humidity; RF: rainfall. MA: moving average

CART models showed the optimal trees had four terminal nodes for Jiuquan, six terminal nodes for Lanzhou and three terminal nodes in Tianshui, respectively (Fig. 6). CART revealed that the optimal regression trees for three study sites are very different. In Jiuquan (Fig. 6), three factors were found to be determinants of weekly HFMD incidence: AT, RH (MA = 10) and MaxT (MA = 5). The overall mean weekly incidence of HFMD was 8.97. The most important splitting factor was AT, with a threshold values of 20 °C. RH (MA = 10) and MaxT (MA = 5) were included as the second and third split in the right branch of the tree, with threshold value of 38% and 27 °C respectively. Thus, the right branch of the tree showed that mean weekly HFMD incidence increased 4.73-fold (to mean weekly HFMD incidence of 42.42 relative to an overall incidence of 8.97) when AT ≥ 20 °C, when RH (MA = 10) < 38%, and when MaxT (MA = 5) ≥ 27 °C.

AT and RH were key determinants of HFMD transmission in Lanzhou. The overall mean weekly incidence of HFMD was 12.31 (Fig. 6). The most important splitting factor was AT, with a threshold value of 17 °C. RH (MA = 10) was involved as the determinants of the rest of nodes. Thus, the right branch of the tree showed that the mean weekly HFMD incidence increased 5.01-fold (to mean weekly HFMD incidence of 61.69 relative to an overall incidence of 12.31) when AT ≥ 17 °C, and when RH between 39% and 44%.

In Tianshui, two factors were found to be determinants of weekly HFMD incidence: AT and RH (MA = 12). The overall mean weekly incidence of HFMD was 3.71. AT was also the most important splitting factors, with a threshold value of 16 °C. Meanwhile, RH (MA = 12) was included as the second split of the three. Thus, the right branch of the tree showed that mean weekly HFMD incidence increased 2.61-fold (to mean weekly HFMD incidence of 9.70 relative to an overall incidence of 3.71) when AT of ≥ 16 °C, and RH (MA = 12) of < 65%.

All three optimal trees shared same primary split nodes (AT), with different threshold values (Jiuquan: 20 °C > Lanzhou: 17 °C > Tianshui: 16 °C). RH (MA) was another weather factor involved in all trees. Based the condition of higher AT, higher RH (MA) could decrease the occurrence of HFMD (Jiuqan:38% < Lanzhou: 49% < Tianshui: 65%). CARTs also indicated that only moderate RH has positive effects for HFMD transmission in Lanzhou when AT ≥ 17 °C. MaxT alone was also used in CART in Jiuquan.

## Discussion

This study quantified different responses of HFMD to weather factors in three specifically-chosen, geographical separated and climate different sites in Gansu Province, China. It can be concluded that HFMD responded differently on different weather factors, and the effect also was a non-linear, and interacted association.

Different seasonal patterns of HFMD infection in three study areas were observed. Other than Jiuquan and Lanzhou, Tianshui had no secondary peaks in seasonal components time series. And the primary peaks in Lanzhou came 2 weeks earlier that other two study areas. As previously reported, Kobe (Japan, 2010), Fukuoka (Japan, from 2000 to 2010), Henan (mainland China, from 2008 to 2013), Suzhou (Jiangsu, mainland China), Shandong (mainland China, from 2008 to 2012) also showed similar pattern in the original HFMD infection, but most parts of the mainland China (Zhejiang, from 2008 to 2012; Huainan, Anhui, from 2009 to 2014; Sichuan, from 2010 to 2014; Guangzhou, Guangdong, from 2009 to 2013; Zhejiang, from 2008 to 2012; Jiangsu, from 2009 to 2013), Taiwan (from 1998 to 2005), Korea (EV71, from 2007 to 2012) and Hong Kong (from 2001 to 2009) had secondary peaks in HFMD infection [14, 21–32]. And the secondary peaks occurred mainly in October and November, which was consistent with the current study in Jiuquan and Lanzhou. One plausible reason was that substantial asymptomatic infected children transmitted the disease to their younger siblings or neighbors during summer holiday, which gave the second rise in September and peaked in November [33]. Another potential reasons and causes of this phenomenon could be that the primary weather drivers of HFMD of two peaks could be different.

With a linear view, different weather could have different effect on HFMD epidemics in different sites. Our study showed temperature, including AT, MaxT and MinT, has positive association with HFMD after taking control of seasonal components. Similar findings have been reported in mainland China, Hong Kong and Japan [14, 29, 34, 35]. Additionally, temperature effect on HFMD in each study areas were different, increasing from Jiuquan to Tianshui. That could possible due to different temperature ranges of three areas that increased from Jiuquan to Tianshui, which had consistent trend with temperature effect in three study areas. We also observed that TD’s association with HFMD were positive in Jiuquan and negative in Lanzhou and Tianshui. Liao found that TD had inverse “V” shaped association with HFMD, which decreasing when TD blew 17 °C, and increasing when TD beyond 17 °C [36]. In our study, TD in Jiuquan was greater than Lanzhou and Tianshui, which indicated that TD could be a key weather factors led to difference response of effect of weather on HFMD in three study areas. The present research also demonstrated that RH had similar role like TD in response of weather effect on HFMD. Zhang reported non-linear, “S” shaped association with thresholds of 45% and 85% between RH and HFMD [37]. In our study, Jiuquan with in low range of RH showed no association with HFMD, RH of Lanzhou may lie in the middle range and presented positive association with HFMD. While Tianshui with higher RH modeled the head of “S” cure, which could be a relatively horizontal line. From a locally aspects, RH showed no statistical association with HFMD. Our study also showed that RF was correlated with HFMD negatively, which was consistent with previous study [36, 38]. RH could protect the susceptible children from social gathering activity or contact in public.

From a nonlinear and interacting view, CART provides more clear information on non-linear interaction among weather factors on HFMD. Considering seasonal components had significant influence on tree building and splitting, seasonal components were not including in the CART model. In the present research, we found that AT and RH were the first two important determinants, but the threshold values for three study areas were slightly different. AT threshold values decreased from northwestern Jiuquan to southeastern Tianshui, which was on the contrary to the actual AT trend. One reason for this phenomenon could be attributed to different enteroviruses molecular types composition in three study areas, since subtype of enteroviruses could have different responses facing different environmental temperature. Models variance and adoption could also induce this difference, longer study period range in the future could help to explore more information on this. With the condition of AT ≥ 20 °C and RH(MA = 10) < 38%, MaxT can still statistically increase the HFMD incidence in Jiuquan, which indicated MaxT also play an important role in Jiuquan. CART tree in Lanzhou also indicated that with the condition of AT ≥ 17 °C, lower RH could increase HFMD occurrence, but it better if RH was higher than 39%.

This study provides novel insights into the different response of impact of weather on HFMD in three study areas. Firstly, study areas were specifically designated, geographical separated and climate different study areas, which made it easy to observe the different response under different weather environment through horizontal comparison. Secondly, we compared and the association between weather factors and HFMD among three study areas. Finally, the association was both measured through linear and nonlinear ways.

Some limitations of this study should also be acknowledged. Firstly, this study designed and collected weekly weather factors of each study areas, so it’s possible that the representativeness of the weather factors varies among study areas. Fortunately, weather stations and population distributed similarly in the whole study areas and gathered more like in urban and suburb, which can narrow this limitation into a more considerable situation. Secondly, measurement and information bias are possible in this ecological study. For example, Jiuquan has more areas than other two study areas, integrated weekly weather factors can only partially represent the whole region. HFMD cases with mild symptoms may be underreported in the surveillance system. Finally, other potential confounding variables such as social factors, enterovirus type, behavioral information and other possible weather factors were not available for the current study.

## Conclusions

Main peaks of HFMD in Lanzhou came 2 weeks earlier than that in Jiuquan and Tianshu. Tianshui had no clear secondary peak in seasonal patterns, which should be further studied. Impact of weather on HFMD in three areas is similar but their thresholds are different. Average temperature is the primary weather factor in three areas, more sensitive in southeast Tianshui, compared with northwest Jiuquan. Relative humidity also plays a non-linear interacting relationship with average temperature on HFMD.

Although many studies have investigated the association between weather and HFMD, our study indicated that the suitable environment for HFMD transmission varies from place to place. Warning models should be built based on local environment. Weather thresholds from CART model can provide a clue to develop HFMD management strategies and public health interventions.

## Abbreviations

CART: Classification and regression tree
CCF: Cross correlation function
GLM: Generalized linear model
HFMD: Hand foot mouth disease
MA: Moving average
